# Adding hydroxyurea in combination with ruxolitinib improves clinical responses in hyperproliferative forms of myelofibrosis

**DOI:** 10.1002/cam4.2147

**Published:** 2019-04-17

**Authors:** Novella Pugliese, Claudia Giordano, Davide Nappi, Luigiana Luciano, Claudio Cerchione, Mario Annunziata, Beniamino Casale, Elena Crisà, Maria Rosaria Villa, Luca Pezzullo, Maria Iovine, Marco Picardi, Francesco Grimaldi, Fabrizio Pane, Vincenzo Martinelli

**Affiliations:** ^1^ Department of Medicine and Surgery Hematology and Hematopoietic Stem Cell Transplant Center University of Naples Federico II Naples Italy; ^2^ Hematology Unit Istituto Scientifico Romagnolo per lo Studio e la Cura dei Tumori (IRST) IRCCS Meldola Italy; ^3^ Hematology Unit A.O.R.N. Antonio Cardarelli Hospital Naples Italy; ^4^ Hematology Unit AO Ospedali dei Colli Napoli, PO Monaldi Naples Italy; ^5^ Hematology Division Università degli Studi di Torino Turin Italy; ^6^ Hematology Unit Ospedale Ascalesi Naples Italy; ^7^ Hematology and Hematopoietic Stem Cell Transplant Center A.U.O. San Giovanni di Dio e Ruggi D'Aragona Salerno Italy; ^8^ Hematology Unit AORN Sant'Anna e San Sebastiano Caserta Caserta Italy

**Keywords:** hydroxyurea, Janus kinase (JAK) inhibitors, primary myelofibrosis, ruxolitinib

## Abstract

Ruxolitinib, an orally bioavailable and selective inhibitor of Janus kinase 1 (JAK1) and JAK2, significantly reduces splenomegaly and disease‐related symptoms in patients with myelofibrosis (MF). However, no clear survival benefit has been demonstrated, which may in part reflect suboptimal drug exposure related to lower dosages needed to minimize hematological toxicity, specifically cytopenias. Furthermore, the optimal management of specific conditions such as leukocytosis or thrombocytosis in patients under ruxolitinib therapy is still undefined. In these cases, combining ruxolitinib with a cytoreductive agent like hydroxyurea might improve hematological response. This observational multi‐center study enrolled 20 adult patients with intermediate‐ or high‐risk primary MF, post‐ polycythemia vera MF, or postessential thrombocythemia MF with hyperproliferative manifestations of the disease and WBC and/or platelet counts not controlled by ruxolitinib therapy. The patients received treatment with a combination of ruxolitinib and hydroxyurea. A clinical response of any type was obtained in 8 patients (40%) during ruxolitinib monotherapy and in 17 patients (85%) during ruxolitinib‐hydroxyurea combination (*P *=* *0.003). After a median duration of 12.4 months of combination therapy, 16/20 patients had a hematological response; 14/17 patients who had started combination therapy to control WBC count and 2/3 who started in order to reduce platelets count. The number of patients requiring ruxolitinib dosage reduction or discontinuations was lower during combination therapy and, at the end of follow‐up the median ruxolitinib dose was increased in 50% of patients. In conclusion, the combination of hydroxyurea with ruxolitinib yielded a high clinical response rate and increased ruxolitinib exposure in patients with hyperproliferative forms of MF.

## 1 INTRODUCTION

Myelofibrosis (MF) is a clonal myeloproliferative neoplasm (MPN) characterized by bone marrow fibrosis and extramedullary hematopoiesis, primarily manifested as splenomegaly.^1^, ^2^, ^3^ The clinical features and the evolution of MF are highly variable and include progressive anemia, leukopenia or leukocytosis, thrombocytopenia or thrombocytosis, and symptomatic splenomegaly.^4^

In the majority of patients with MF, symptoms feature and burden together with the IPSS score^5^, ^6^ are the main determinants of treatment choice. Therapeutic options included best supportive care/transfusion support, cytoreduction with hydroxyurea or interferon, use of erythropoietin stimulating agents, immunomodulatory drugs such as thalidomide and lenalidomide, danazol, radiotherapy, and splenectomy.^5^, ^6^ Among these, hydroxyurea has been shown to have a significant impact on the reduction in spleen and liver size, constitutional symptoms, pruritus, and bone pain, and to control leukocytosis and thrombocytosis, with a reported overall response rate of 40% and a median duration of response of 13.2 months.^7^

However, after the discovery of dysregulation of the Janus kinase (JAK) pathway, most commonly JAK2 V617F mutation, in over half of MF patients,^8^, ^9^ targeted therapies with JAK inhibitors have demonstrated improvements in splenomegaly, burden of symptoms, and a potential gain in overall survival.^10^, ^11^

Two pivotal Phase III studies, COMFORT I and II, demonstrated that the potent and selective inhibitor of JAK1 and JAK2, ruxolitinib, had great efficacy in reducing splenomegaly and disease‐related symptoms, however, without a clear demonstration of benefits in terms of survival.^10^, ^11^ Afterwards, longer term follow‐up and pooled analysis of COMFORT I and II supported a survival benefit for MF patients who received treatment with ruxolitinib.^12^, ^13^ It has been suggested that the lack of survival benefit observed in the pivotal studies may reflect the necessity for dosage modulations, mostly due to hematological toxicity, but also to reduced control of disease, resulting in a reduced overall drug exposure.^14^ Noteworthy, drug discontinuations were associated with cytopenia, loss of spleen response and disease progression expressed by leukocytosis.

For these reasons, a combination of ruxolitinib and a cytoreductive agent such as hydroxyurea may be hypothesized to improve hematological response in the “hyperproliferative” forms of the disease, characterized by constitutional symptoms, marked splenomegaly, leukocytosis, and/or thrombocytosis. However, to date, there are only anecdotal reports about the value and efficacy of the addition of hydroxyurea to ruxolitinib treatment.^15^, ^16^, ^17^

Although the primary target of ruxolitinib therapy in MF is to control spleen‐ and disease‐related symptoms, the recent consensus report of the International Working Group (IWG) and the European LeukemiaNet (ELN) also includes WBC and platelet count control to below the upper limit of normal among their revised response criteria for treatment response for MF.^18^, ^19^

To this end, in this study, patients affected by MF and with leukocytosis and/or thrombocytosis uncontrolled while on ruxolitinib monotherapy were moved to combination treatment with ruxolitinib plus hydroxyurea.

## 2 METHODS

This retrospective multicenter case series, collected on a survey basis, was conducted from April 2012 to April 2017, and 20 adult patients (≥18 years of age) with a confirmed diagnosis of primary MF (PMF), post‐polycythemia vera (PPV‐MF), or post‐essential thrombocythemia (PET‐MF) with hyperproliferative manifestations of the disease were enrolled. Patients who were scheduled to receive hydroxyurea in combination with ruxolitinib at the time when WBC and/or platelet count were in the upper to normal range (WBC > 10.0 × 10^9^/L and platelets > 400 × 10^9^/L),^18^, ^19^ while on ruxolitinib monotherapy, regardless of symptom improvements and spleen response obtained while on treatment with ruxolitinib, were included in this analysis. Patients at the time of starting ruxolitinib had intermediate or high‐risk disease according to the IWG for MF.^20^

### 2.1 Treatment schedule

All patients evaluated had received ruxolitinib orally twice daily continuously at a starting dose based on baseline platelet count: 5 mg twice daily bis in die (BID), platelets 50 to <100 × 10^9^/L; 15 mg BID, 100‐200 × 10^9^/L; or 20 mg BID, >200 × 10^9^/L according to the dosage schedule established by the drug data sheet.^21^ If platelet and neutrophil count were not controlled by therapy and/or treatment efficacy was insufficient, ruxolitinib doses were modified by 5 mg BID increments (up to 25 mg BID).^21^

The starting dose of hydroxyurea during combination therapy was based on clinician choice and modulated on the basis of WBC and platelet count and on the maximum ruxolitinib dose tolerated by each individual patient. The initial doses for combination therapy were classified as follow: low doses, that is, low ruxolitinib (<10 mg BID)/low hydroxyurea (<1000 mg/daily); high doses, ie ruxolitinib (≥10 mg BID)/high hydroxyurea (≥1000 mg/daily) and intermediate doses, that is, low ruxolitinib/high hydroxyurea or high ruxolitinib/low hydroxyurea. Doses of both hydroxyurea and ruxolitinib were modulated according to efficacy on disease symptoms and adverse events. The combined treatment was continued unless significant toxicity was observed or WBC and/or platelet control was obtained. Discontinuation of ruxolitinib was defined as a period of time in which patients needed treatment interruption due to side effects. Treatment could be resumed after recovering. Side effects were assessed and graded according to National Cancer Institute Common Terminology Criteria for Adverse Events version 4.03.^22^

### 2.2 Response assessment

Patient responses were defined according to ELN criteria.^18^, ^19^ Response were stratified on the basis of initial combination doses.

Symptoms were evaluated by using MPN Symptom Assessment Form questionnaires,^23^ and a reduction in ≥50% of symptoms was considered as a response to treatment. Spleen size was assessed by physical examination. A baseline splenomegaly palpable at 5‐10 cm from the left costal margin (LCM) that became nonpalpable or a baseline splenomegaly palpable >10 cm from the LCM that decreased by ≥50% was considered as a response to treatment.^18^, ^19^ In terms of hematological parameters, a WBC count of between 4 and 10 × 10^9^/L and a platelet count of between 100 and 400 × 10^9^/L were considered to be a complete response.^18^, ^19^

## 3 RESULTS

Twenty patients with PMF, PPV, PET, treated at 6 hematology divisions, were included and retrospectively analyzed. All patients had received ruxolitinib as monotherapy for a median duration of 15.9 months (range 1.6‐181 months) from MF diagnosis. The baseline characteristics of the patients when ruxolitinib was started are summarized in Table 1. Nineteen patients had received a median of one prior therapy (range 1‐4) for MF. Nineteen patients (95%) had been treated with hydroxyurea as a single agent, 5 patients (25%) had received corticosteroid treatment, 2 patients (10%) had received pegylated interferon alpha, and 1 patient (5%) had received danazol. The starting dose of ruxolitinib as monotherapy was 20 mg BID for 8 patients (40%), 15 mg BID for 7 patients (35%), 10 mg BID for one patient (5%), 5 mg BID for 4 patients (20%).

**Table 1:** Baseline clinical characteristics of the patients at the time of starting ruxolitinib (n = 20)

Variable	
Sex	
Female	5 (25)
Male	15 (75)
Age, years	
Median (range)	64 (36‐82)
Myelofibrosis subtype	
Primary myelofibrosis	7 (35)
Post‐polycythemia vera myelofibrosis	6 (30)
Postessential thrombocythemia myelofibrosis	7 (35)
IPSS risk status	
Intermediate‐1	3 (15)
Intermediate‐2	7 (35)
High	10 (50)
Hemoglobin—g/dL	
Median (range)	10.9 (7.5‐15.3)
Pts with anemia^a^ (%)	13 (65)
Platelet count—×10^9^/L	
Median (range)	212 (63‐800)
Pts with thrombocytosis^a^ (%)	5 (25)
WBC count—×10^9^/L	
Median (range)	18.6 (4.9‐58.4)
Pts with leukocytosis^b^ (%)	17 (85)
Splenomegaly	
Present	20 (100)
Spleen length	
Median (range)	18 (8.9‐24)
Janus kinase 2 V617F	
Mutated	18 (90)
Prior treatment	
Yes	19 (95)

Anemia not attributed to a comorbid condition.

a Leukocytosis > 11 × 10^9^/L.

b Thrombocytosis ≥ 450 × 10^9^/L.

At the starting time of the study, 17 patients (85%) started hydroxyurea treatment due to lack of WBC control, whereas the remaining three patients (15%) started for the lack of platelet control, two of these had platelet counts of 2000 × 10^9^/L (Table 2). Among patients who started combination therapy due to leukocytosis, four patients had platelet counts below 100 × 10^9^/L. On the other hand, among the three patients who started combination therapy because of unsatisfactory platelet control, all patients showed a WBC count below 10 × 10^9^/L. The addition of hydroxyurea was performed after a median time of ruxolitinib monotherapy of 6.5 months (range 1‐49.6 months). The starting doses of the two drugs at the beginning of the combination therapy were as follow: hydroxyurea 500 mg daily plus ruxolitinib 5 mg BID for 6 patients (30%), hydroxyurea 1000 mg daily plus ruxolitinib 5 mg BID for 2 patients (10%), hydroxyurea 500 mg daily plus ruxolitinib 10 mg BID for 3 patients (15%), hydroxyurea 1000 mg daily plus ruxolitinib 10 mg BID for 1 patient (5%), hydroxyurea 1000 mg daily plus ruxolitinib 15 mg BID for 4 patients (20%), hydroxyurea 1000 mg daily plus ruxolitinib 20 mg BID for 2 patients (10%) and hydroxyurea >1000 mg daily plus ruxolitinib 20 mg BID for the remaining 2 patients (10%). Overall, six patients received low initial doses, five patients received intermediate doses and the remaining 9 patients received high combination doses.

**Table 2:** Patient characteristics at the start of combination therapy

Variable	Reason for adding hydroxyurea	
	Leukocytosis control (n = 17)	Thrombocytosis control (n = 3)
Time of ruxolitinib monotherapy		
Median (range)	18 (1‐49.6)	3.74 (3‐13)
Hemoglobin—g/dL		
Median (range)	11 (6.7‐13)	9.2 (8.5‐10.2)
Platelet count—×10^9^/L		
Median (range)	214.5 (27‐405)	2000 (558‐2000)
White blood cell count—×10^9^/L		
Median (range)	21.4 (16.1‐69.9)	7.0 (2.3‐8.0)
Splenomegaly		
Present	17 (100)	3 (100)
Hydroxyurea starting dose, g		
Median (range)	0.5 (0.5‐1.0)	2.0 (0.5‐3.0)
Time of combination therapy		
Median (range)	9.5 (4‐44)	6.0 (4‐18)

The median time of the combination therapy was 14.5 months (range 2‐195).

**Figure 1:**
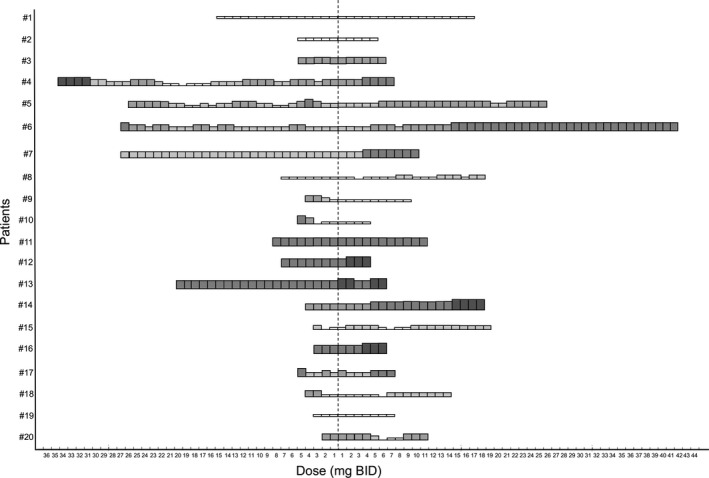
Dose profiles of ruxolitinib in 20 patients treated with ruxolitinib monotherapy and ruxolitinib in combination with hydroxyurea

While on ruxolitinib monotherapy, 10 patients (50%) needed a dose reduction due to hematological toxicity and three patients temporarily discontinued ruxolitinib due to severe thrombocytopenia (15%), as shown in Table 3 and in Figure 1.

**Table 3:** Adverse events observed in patients during the ruxolitinib monotherapy and the combination treatment

	Ruxolitinib monotherapy (n = 20)		Ruxolitinib plus Hydroxyurea (n = 20)	
Hematological toxicity^a^	All grades	Grade 3 or 4	All grades	Grade 3 or 4
Thrombocytopenia	6 (30)	3 (15)	8 (40)	2 (10)
Anemia	11 (55)	2 (10)	7 (35)	2 (10)
Neutropenia	2 (10)	—	1 (5)	—
Ruxolitinib discontinuation	3 (15)		1 (5)	
Ruxolitinib dose reduction	10 (50)		2 (10)	
Hydroxyurea discontinuation	—		5 (25)	
Hydroxyurea dose reduction	—		8 (40)	

Unless otherwise indicated, data are number of patients, with percentage in parentheses.

a Hematologic abnormalities are based on laboratory values. The data shown are for the events of the worst grade during the study, regardless of whether this grade was a change from the baseline grade.

The combined ruxolitinib/hydroxyurea therapy was very well tolerated, and the hematological toxicities mostly occurred in the first 8‐12 weeks of treatment, and were generally manageable with dose reductions and/or supportive treatment. In eight patients the ruxolitinib daily doses increased (mean increase = 5.1 mg BID), in 9 the dose was unmodified despite the addition of hydroxyurea, and only in three cases the ruxolitinib dose had to be reduced (mean reduction = 3.4 mg BID). Overall, the mean daily dose of ruxolitinib administered to the whole cohort of patients increased by 1.8 mg BID (*P *=* *0.013), and only one patient had to discontinue ruxolitinib, due to severe thrombocytopenia (Figure 1). The dose of hydroxyurea was down‐modulated in eight patients (40%) according to the individual hematological compliance to the treatment. Nonhematological adverse events were not observed.

After a median of 11.1 months (range 2‐35 months) of ruxolitinib monotherapy, 6 patients obtained a clinical improvement in MF‐associated symptomatic burden, consisting of improvement of symptoms and spleen improvement in 5 of them and improvement of symptoms in the remaining patient. After a median 12.4 months (range 4‐44 months) of combination therapy, of the 17 patients who started the combination therapy to control WBC count, 14 (82.3%) obtained a WBC response, whereas, among the three patients who started hydroxyurea in association with ruxolitinib in order to reduce platelets count, 2 (66.6%) achieved a platelet response. Data relating to response according to dose of the combination therapy are reported in Table 4.

**Table 4:** Clinical and hematological response with ruxolitinib monotherapy and after combination therapy for all patients, and according to combination therapy doses

	Ruxolitinib monotherapy (n = 20)	Ruxolitinib plus hydroxyurea			
		All patients (n = 20)	Low doses (n = 6)	Intermediate doses (n = 5)	High doses (n = 9)
Duration of treatment, months, median (range)	11.1 (2‐35)	12.4 (4‐44)	10.5 (4‐19)	9 (5‐19)	10 (4‐44)
Clinical response of any type	8/20 (40.0)	17/20 (85.0)	5 (83.3)	4 (80.0)	8 (88.9)
Symptoms response	6/20 (30.0)	12/20 (60.0)	2 (33.3)	4 (80.0)	6 (66.7)
Spleen response	5/20 (25.0)	9/20 (45.0)	1 (16.7)	3 (60.0)	5 (44.4)
White blood cell response	—	14/17 (82.3)	4/6 (66.7)	4/5 (80.0)	6/6 (100.0)
Platelet response	—	2/3 (66.7)	—	—	2/3 (66.7)

Unless otherwise indicated, data are number of patients, with percentage in parentheses. Low doses: low ruxolitinib (<10 mg BID)/low hydroxyurea (<1000 mg/daily); intermediate doses: low ruxolitinib/high hydroxyurea or high ruxolitinib/low hydroxyurea; high doses: ruxolitinib (≥10 mg BID)/high hydroxyurea (≥1000 mg).

In addition, 12 patients of the whole study cohort had clinical improvement in MF‐associated symptomatic burden, with 9 of them showing spleen and symptoms response and the remaining three only spleen responses (Table 4). Of note, all eight patients in whom ruxolitinib dose was increased had at least one type of drug‐related clinical response.

## 4 DISCUSSION

We describe a retrospective multi‐center case series carried out to investigate the role of the combination of hydroxyurea with ruxolitinib in adult patients with hyperproliferative forms of MF.

Ruxolitinib, the first JAK inhibitor approved for the treatment of MF, is generally well tolerated, with thrombocytopenia as the dose‐limiting toxicity; evidence from clinical trials support the efficacy of ruxolitinib for hyperproliferative manifestations of MF.^10^, ^11^, ^12^, ^21^, ^24^, ^25^, ^26^, ^27^ Reduction in spleen size and control of symptoms are usually rapid and durable, but also drug‐ and dose‐dependent, given that discontinuing or reducing the dose of ruxolitinib is followed by rapid increase in spleen size and reappearance of symptoms. Although hydroxyurea is widely used to attenuate hyperproliferative manifestations related to MF, there are few well‐designed studies evaluating hydroxyurea benefits in patients with MF.^7^, ^28^, ^29^ In a group of 40 patients with PMF, Martinez‐Trillos et al. showed significant response rates, with reductions in constitutional symptoms (55%), symptomatic splenomegaly (45%), thrombocytosis (40%), and leukocytosis (28%). Accentuation of anemia was the most common adverse event and was observed in almost half of the patients.^7^

To the best of our knowledge, this is the first retrospective case series to determine the feasibility and efficacy of combining ruxolitinib with hydroxyurea in a particular setting of MF patients affected by hyperproliferative disease. In this case series, patients in which ruxolitinib monotherapy was unable to reduce WBC and platelet within normal ranges (WBC ≤ 10.0 × 10^9^/L or platelet ≤ 400 × 10^9^/L)^18^, ^19^ received the combination in order to overcome hematological resistance and to better control disease‐related symptoms and splenomegaly.

Overall, in our study, ruxolitinib plus hydroxyurea induced a hematological response in all but four patients, independently of initial combination doses. In addition, this combination therapy induces a higher rate of either spleen and symptoms responses than those observed after the ruxolitinib monotherapy (spleen response was obtained in 5 patients receiving ruxolitinib monotherapy vs 8 receiving the combination; symptoms response was reached in 6 vs 12 patients treated with ruxolitinib monotherapy and in combination with hydroxyurea, respectively). This slight difference is not statistically significant and we cannot state with certainty that the improvement in the spleen response depends on the combination or on the doses of each drug (which, in the case of ruxolitinib, increases with the combination therapy). Indeed, the rate of spleen and symptoms response was lower for the patients on low doses compared with those on intermediate and high doses. Even if this difference was not statistical significant, it could suggest that spleen and symptoms responses are also dose dependent during combination therapy. On the other hand, it cannot be excluded that a late response would, in any case, have been observed with monotherapy.

The addition of hydroxyurea to ruxolitinib did not result in lower compliance to the latter drug. Instead, we were able to increase the ruxolitinib dose in 8 patients, and only three patients had to reduce the dose of this drug. Only 2 patients (10%) experienced severe thrombocytopenia, although only 1 patient needed temporary drug discontinuation. For the remaining patients, platelet counts initially decreased and subsequently stabilized at a new steady state. On the other hand, it is plausible that the increase in overall treatment intensity may be the main determinant for the better outcome of the therapy (all patients with ruxolitinib dose increase had experienced improvement). Indeed, our results indicate that combination of ruxolitinib and hydroxyurea results in enhanced efficacy of the treatment not only regarding hematological responses, observed in 16 out 20 patients, but also in terms of clinical responses, that is, splenomegaly and control of symptoms, observed in 12 out the 20 patients. In this regard, it should be underlined that both leukocytosis and thrombocytosis are associated with an enhanced risk of cardiovascular events.^30^, ^31^, ^32^ In addition, the combination of hydroxyurea with ruxolitinib, besides giving better clinical control of the disease, allowed the dose of the latter drug to be increased and the duration of its administration to be prolonged. Long‐term follow‐up from COMFORT‐II indicates that prolonged exposure to ruxolitinib therapy has a positive impact on the probability of survival of patients. Indeed, median overall survival was not reached in the ruxolitinib arm, vs 4.1 years in the best available therapy (BAT) arm, with a 33% reduction in risk of death with ruxolitinib vs BAT by intent‐to‐treat analysis.^12^

Our study has several limitations which must be pointed out. Firstly, it is a multi‐center retrospective study with a small number of patients, which limits statistical power. Hence, our findings need to be validated in a large prospective trial. In particular, the stratification of response on the basis of combination doses does not allow a statistical analysis. Secondly, although all centers used the same criterion regarding platelet and leukocyte control to start the combination therapy, the starting dose of hydroxyurea and the dose used during the combination period were based on clinician choice (whereas the ruxolitinib dosage was modulated according to the dosage schedule established by the drug data sheet^19^). Therefore, a prespecified protocol was not used for the hydroxyurea dose. Indeed, to the best of our knowledge, there are no guidelines on the initial dose of hydroxyurea in patients with MF. Thirdly, WBC and platelet count control below the upper limit of normal as response criteria for MF are not recommended for use in the clinical practice, due to the lack of well documented benefit to outcome. Even if we can define with certainty that leukocytosis represents an unfavorable prognostic factor in patients with MF and therefore controlling it could be expected to improve the outcome of the patients, there is no unequivocal interpretation of the prognostic value of thrombocytosis or of any additional risk it imposes.^20^, ^30^, ^31^, ^32^, ^33^, ^34^ On the other hand, 2 of the 3 patients who started combination therapy for failure to control the platelet count had platelet counts above > 1500 × 10^9^/L; this value unquestionably represents a condition of hemorrhagic risk that requires a cytoreductive therapy for control of platelets.^30^, ^35^

In conclusion, the combination of ruxolitinib with hydroxyurea is an effective and relatively well‐tolerated therapy for the hyperproliferative manifestations of MF, independently of combination dose. Accentuation of the thrombocytopenia, usually manageable with drug reduction or short discontinuation of combination therapy, is the most frequent side effect of treatment. The durability of the responses is variable but can be long‐lasting. Nevertheless, given the results of our study, this association seems an effective and safe therapy for MF, and in particular for hyperproliferative forms of MF, hence, our findings need to be validated in a prospective and well‐controlled large trial.

## CONFLICT OF INTEREST

The authors declare that they have no competing interests.

## AUTHOR CONTRIBUTIONS

All authors contributed to the writing and/or revision of the manuscript, and we have all read and approved the submission of this manuscript.

## ETHICS STATEMENT

Patients were asked to sign a consent form, approved by the local ethics committee of each participating institution, according to the requirements of the Helsinki declaration.
